# Comparative effectiveness of pelvic floor muscle training, mirabegron, and trospium among older women with urgency urinary incontinence and high fall risk: a feasibility randomized clinical study

**DOI:** 10.1186/s40814-023-01440-w

**Published:** 2024-01-04

**Authors:** Steve R. Fisher, Alejandro Villasante-Tezanos, Lindsay M. Allen, Monique R. Pappadis, Gokhan Kilic

**Affiliations:** 1 Department of Physical Therapy, University of Texas Medical Branch at Galveston, Galveston, USA; 2 Office of Biostatistics, University of Texas Medical Branch at Galveston, Galveston, USA; 3 Department of Population Health and Health Disparities, University of Texas Medical Branch at Galveston, Galveston, USA; 4 Department of Obstetrics and Gynecology, University of Texas Medical Branch at Galveston, Galveston, USA

**Keywords:** Overactive bladder, Falls, Urinary incontinence

## Abstract

**Background:**

Untreated, urgency urinary incontinence (UUI) and overactive bladder (OAB) can precipitate a vicious cycle of decreasing physical activity, social isolation, fear of falling, and falls. Structured behavioral interventions and medications are common initial treatment options, but they elicit their effects through very different mechanisms of action that may influence fall-related outcomes differently. This study will determine the feasibility of conducting a comparative effectiveness, three-arm, mixed methods, randomized clinical trial of a behaviorally based pelvic floor muscle training (PFMT) intervention versus two recent drug options in older women with UUI or OAB who are also at increased risk of falling.

**Methods:**

Forty-eight women 60 years and older with UUI or OAB who screen positive for increased fall risk will be recruited through the urogynacology and pelvic health clinics of our university health system. Participants will be randomly assigned to one of three 12-week treatment arms: (1) a course of behavioral and pelvic floor muscle training (PFMT) provided by physical therapists; (2) the beta-3 agonist, mirabegron; and (3) the antimuscarinic, trospium chloride. Study feasibility will be established through objective metrics of evaluability, adherence to the interventions, and attrition. We will also assess relevant measures of OAB symptom severity, quality of life, physical activity, incident falls, and concern about falling.

**Discussion:**

The proposed research seeks to ultimately determine if linkages between reduction in UI symptoms through treatment also reduce the risk of falling in this patient population.

**Trial registration:**

NCT05880862. Registered on 30 May 2023.

## Background

Urinary urgency (*sudden overwhelming desire to void that is difficult to defer*), frequency, and nocturia (*frequent nighttime urination*) are the most common urinary problems in women [1]. Collectively, these symptoms describe overactive bladder syndrome (OAB) [2]. Urgency urinary incontinence (UUI) is defined as a sudden, intense urge to urinate followed by an involuntary loss of urine [2]. The prevalence of these stigmatizing conditions among older women living in the community is estimated to be > 30% [1], but this estimate is likely imprecise; fewer than half are believed to report their bladder control problems to a healthcare provider [3]. Long-term effects include social isolation, loss of independence, a debilitating fear of falling, and injurious falls [4, 5].

The association between urinary problems and falls has been attributed to several factors [6–9]. These include an increase in diurnal and nocturnal voiding [6, 7, 10]; the diverting of cognitive attention in the midst of urgency [11, 12]; and self-imposed activity limitations [13] which can precipitate deconditioning, fear or falling, and actual falls [14]. Side effects associated with some commonly used medications are likely contributors as well, such as impaired cognition, drowsiness, blurred vision, and dizziness [15, 16]. A recent scoping review on UUI treatment and fall risk found serious gaps in the literature regarding research designed to examine linkages between the two, especially among frail older adults [17].

It remains unknown, however, if current treatments for UUI and OAB, including conservative and pharmacological treatments, either reduce or increase the risk of falling in older people [18]. Initial non-surgical treatment options vary significantly in their mechanisms of action and potential impact on fall risk. Behavioral interventions, such as pelvic floor muscle training (PFMT), are performed by physical therapists (PTs) or nurse practitioners (NPs) and focus on strengthening the pelvic floor and changing behaviors that influence urinary symptoms. Medications primarily target detrusor muscle contractions in the bladder. The introduction of the B3-adrenergic receptor agonist, mirabegron, and the antimuscarinic, Trospium, has broadened available medical management options for UUI and OAB considerably. Clinical efficacy is comparable to the traditionally used anticholinergics but with fewer cholinergic side effects [19]. This is important because anticholinergic burden is correlated with cognitive decline and Alzheimer’s disease [20]. These newer drugs are now the preferred medications for older adults [21].

Large gaps remain in the literature on UUI and OAB care regarding comparisons of individual interventions, particularly among specific patient subgroups [22]. In the few studies that have compared behavioral to pharmacologic interventions, traditional anticholinergic drugs were the comparators and the participants were generally younger, higher functioning, and not from underrepresented racial and ethnic minorities [23–26]. There is a need for more targeted interventions on specific subpopulations of women with UI as well as a better understanding of the bidirectional mechanisms that link UI to physical activity and other geriatric syndromes such as falls [27]. Therefore, this feasibility study will test the central hypotheses that (i) a randomized pilot multi-arm clinical trial comparing PFMT to the two most recent pharmacologic agents for UUI or OAB in older women at high risk of falling is feasible, and (ii) treatment approach can influence both UI and fall-related outcomes in this patient population.

## Methods

### Study design

This is a pilot, single-blind, three-arm, mixed methods, randomized clinical trial. The delivery of our comparators will closely mirror their provision in current clinical practice using schedules and dosages that are recognized by Medicare and most insurance. Participants (16 in each arm) will be randomly assigned (1:1:1) to the three treatment groups and assessed quantitatively and qualitatively at baseline, 12 weeks (primary endpoint), and 6 months.

The Centers for Disease Control and Prevention (CDC) “3 Key Questions” fall screening tool will be used to identify women at high risk for falls. The “3 Key Questions” are part of the CDC’s Stopping Elderly Accidents, Deaths, and Injuries (STEADI) initiative to facilitate fall risk identification and management in primary care [28]. Women who answer “yes” to any of the following three questions are considered to be at increased risk of falling [29]: (1) Have you fallen in the past year? (2) Do you feel unsteady when standing or walking? and (3) Do you worry about falling? CONSORT reporting guidelines were used in the development of this study [30].

### Setting

Recruitment will occur primarily through the outpatient Urogynacology and Pelvic Health clinics at our university teaching hospital. The recruitment, screening, and enrollment workflow was developed collaboratively by investigative team members. The “3 Key Questions” are included among the intake forms for all patients seen through the Division of Urogynecology. The form is completed during the patient’s scheduled clinic visit.

### Participants

The criteria are designed to be inclusive of participants who could receive any of the three interventions during regular standard of care.

Inclusion criteria for the study include (1) women aged 60 years or older; (2) able to walk across a small room with or without an assistive device; (3) diagnosed urgency UI, OAB, or Mixed UI (both urgency and stress UI); (4) answered “yes” to one of the items on the 3-Key Questions, questionnaire [28]; (5) a score of ≥ 6 on the *International Consultation on Incontinence Modular Questionnaire—Overactive Bladder* (ICIQ-OAB) instrument — or treatment recommended by the study physician [31]; (6) able to provide her own informed consent; (7) she has tried basic lifestyle modifications for her bladder condition such as scheduled voiding or cutting back on bladder irritants, such as alcohol, caffeine, and/or acidic foods; and (8) has Medicare, Medicaid, or private insurance.

Exclusion criteria include (1) male (their causes of urinary incontinence are often different from women); (2) unstable psychiatric conditions (e.g., psychosis, suicidal) based on history and medical records; (3) nursing home resident; (4) undergoing active treatment with chemotherapy or radiation for genitourinary cancer; (5) neurologic conditions known to contribute to incontinence (multiple sclerosis, Parkinson’s disease, TBI, dementia, and stroke survivors with limited mobility); (6) any new OAB treatments planned during the 6-month study duration (includes medications and/or surgery). botulinum toxin bladder injections for UI in the previous six months or a history of surgically implanted sacral nerve stimulator; (7) taking any other antimuscarinic drugs or digoxin; (8) severe uncontrolled hypertension; and (9) diagnosed glaucoma, myasthenia gravis, or end-stage liver or kidney disease.

### Comparators/interventions

Our comparators are the preferred, initial therapies for the treatment of UUI and OAB in older women: They are (1) a 12-week, 6 visits, outpatient program of PT-delivered behavioral, and PFMT; (2) individually titrated mirabegron, starting at 25 mg daily and increased to 50 mg daily at 6 weeks, during the 12-week intervention period; or (3) a 12-week course of Trospium XL -extended release, 60 mg once daily.

### Pelvic floor muscle training

The effectiveness of PFMT with urge suppression has been established in several placebo-controlled trials using intention-to-treat models, in which mean reductions of UI range from 60 to 80% [23, 26]. The behavioral-PFMT intervention for the proposed trial is based on clinical practice guidelines, behavioral training principles, and findings from exercise adherence literature [32–40]. The PT provider has advanced training in women’s health and treatment of disorders of the pelvic floor.

The PFMT intervention is a comprehensive training program using pelvic floor exercises, delayed voiding, and urge suppression techniques to inhibit or suppress detrusor contractions and reduce urgency, frequency, and incontinence. The program consists of 6, 1-h long sessions over 12 consecutive weeks. An internal pelvic examination is completed on all patients during the initial assessment. If the patient refuses the pelvic exam, services can still be rendered (e.g., surface EMG can be utilized to monitor early muscle activation; project therapists report few women in this age group refuse the internal pelvic exam). Education also includes information on common bladder irritants and instructions in the completion of a bladder voiding diary. Pelvic floor exercises, urge control strategies, and a home program are introduced early [41]. Patients with mixed UI are instructed in techniques designed to control leaks during every day activities such as coughing, lifting, or blowing the nose [42, 43]. As neuromuscular control improves, stronger contractions that may incorporate functional activities are emphasized [44–46]. Intensity of exercises is increased through increased repetition and longer endurance holds, as well as progression to against gravity positions (e.g., in standing) from the more gravity-eliminated (e.g., supine) positions.

### Medications

Participants randomized to the medications arms will receive either (i) individually titrated mirabegron, starting at 25 mg daily and increased to 50 mg daily at 6 weeks; at the scheduled 6-week visit with the physician, blood pressure and heart rate will be monitored to determine dose continuation or dose escalation to the 50 mg dosage; or (ii) 60 mg trospium chloride extended release once daily for the duration of the 12-week intervention. If side effects are not tolerable with either medication, it will be discontinued, and she will continue in the study for all outcomes.

*Mirabegron* is the only FDA-approved beta-3 agonist for the treatment of OAB with UI. Recent multi-center trial evidence on use of mirabegron compared to placebo in older adults (*n* = 888) showed improvement in mean micturitions over 24 h (− 0.7, 95% CI − 1.0, − 0.3, *p* < 0.001) and number of UI episodes (− 0.6, 95% CI − 0.8, − 0.3, *p* < 0.001) [47]. In addition to showing efficacy in older adults with OAB and UI, clinical trial data also reported safety and efficacy with use over a 12-week period [48]. The most common treatment emergent adverse events (TEAEs) in mirabegron-treated patients were UTI, headache, and diarrhea. Blood pressure and heart rate were monitored closely without major changes seen in the mirabegron vs. the placebo arm. No mirabegron-related TEAEs occurred, including falls [48].

*Trospium chloride* is an FDA-approved quaternary amine antimuscarinic drug used to treat OAB. It has a low propensity to cross the blood–brain barrier and cause CNS adverse effects such as cognitive dysfunction and minimizes the potential for metabolic drug-drug interactions [49, 50]. Randomized double-blind, multi-center clinical trials comparing trospium to placebo in adults (*n* = 517) have shown significant improvements in the patients’ assessment of treatment efficacy (*p* < 0.001), frequency of voids per day (− 1.9 vs. − 2.7; *p* < 0.001), and UUI episodes per day (− 1.8 vs. − 2.4) [51]. The main complaint was dryness of the mouth. Subgroup analyses of large RCTs on Trospium have supported the efficacy and safety of Trospium in patients 75 and older with OAB and multiple co-morbidities and at risk for drug-drug interactions, impaired cognition, and frailty [52].

### Measures

#### Study measures

This pilot study is designed to gather feasibility information to inform a larger comparative effectiveness trial. Thus, measures include feasibility as well as outcome measures planned for the main study to ensure they can be captured appropriately.

Feasibility will be defined based on (1) the proportion of enrolled participants who are evaluable and (2) the proportion adherent to the interventions. Evaluability is defined as the number of participants completing the baseline and both post-intervention assessments divided by the number enrolled. *Safety* of the interventions: The number of adverse events will be recorded and graded.

*Adherence* will be defined as:

- PFMT arm: clinic-based adherence over the 12-week intervention will be calculated by dividing the number of sessions attended by the total number of scheduled sessions [53] (six sessions are scheduled for the 12-week therapy course). Home-based adherence will be a percentage of adherence to the prescribed home program [54]. Patients complete a home exercise diary each time they perform prescribed exercises. The diaries are taken to every clinic appointment and reviewed by the provider.
- Medication arms: adherence will be calculated as pill counts, i.e., the number of pills dispensed minus the number of pills returned [55]. The number of pills provided to the patient will be documented in study research records. Participants will be asked to note the date/time they take the medication each day on a provided study diary and asked to bring their empty medication containers and any remaining pills with their study diary to all study visits. Diaries will be reviewed at each visit by research staff. In instances where the study physician believes the medication needs to be discontinued or escalated (e.g., due to side effects or unexpected medical events), site research staff will be informed, and the event and its rationale documented. If a participant stops taking prescribed medications altogether, that will be identified during the scheduled contacts taking place every two weeks and documented. Evidence suggests that most patients (> 70%) stop using anticholinergics within 5 months, mostly because of side effects, less data exists for Mirabegron and Trospium.

Acceptability of the interventions: will be assessed qualitatively via semi-structured interviews and quantitatively using the Acceptability of Intervention Measure (AIM), a validated 4-item measure of perceived intervention acceptability.

#### Exploratory measures

Primary exploratory outcomes include validated measures of urgency symptoms and UI-related quality of life. Secondary exploratory outcomes include incident falls, concern about falling, physical functioning, and physical activity. Variation and effect sizes for this specific patient population will be calculated and used to refine the theoretical model and to determine sample sizes for a future fully powered clinical trial. Table 1 shows the schedule of assessment for all exploratory variables and sociodemographic variables.

**Table 1:**
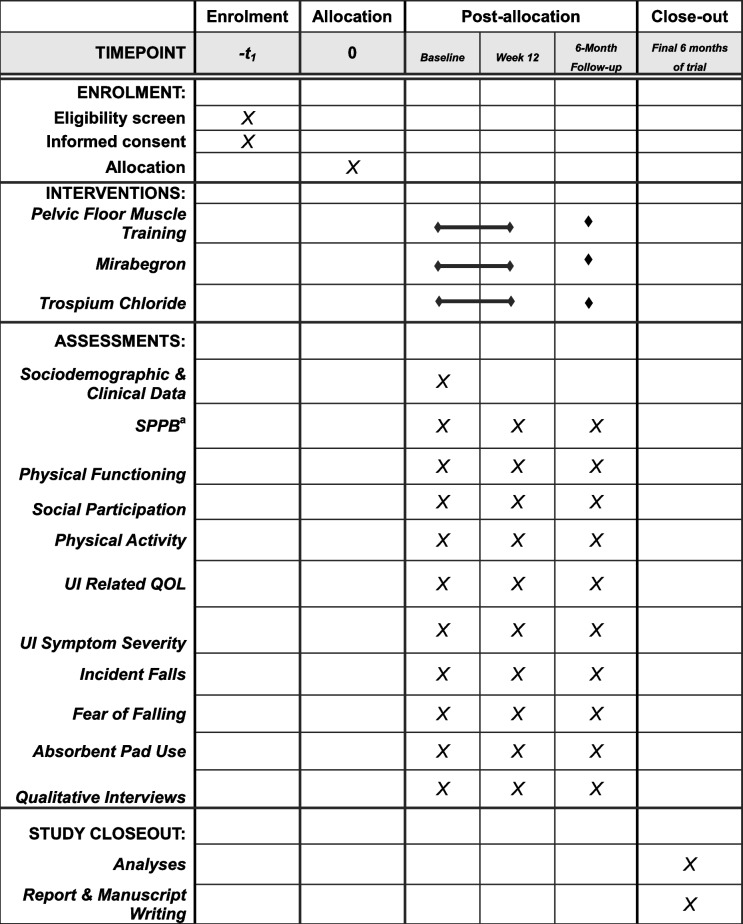
Schedule of enrolment, interventions, and assessments

^a^*SPPB* Short Physical Performance Battery

##### Primary exploratory outcomes

- OAB symptom severity: The International Consultation on Incontinence Modular Questionnaire—Overactive Bladder (ICIQ-OAB) [56]. *The International Consultation on Incontinence Modular Questionnaire—Overactive Bladder (ICIQ-OAB):* The ICIQ-OAB is a patient-reported outcome that captures the self-perceived degree of urinary frequency, urinary urgency (rushing to the bathroom), and urge incontinence (leakage) as well as symptoms related to nocturia (nighttime urination) [56]. The 4-item scale ranges from 0 to 16 with greater values indicating increased symptom severity. Bother scales associated with each question are not incorporated in the overall score but indicate the impact of individual symptoms.
- UI-related quality of life: International Consultation on Incontinence Questionnaire Lower Urinary Tract Symptoms Quality of Life (ICIQ-LUTSqol) [57]. The ICIQ-LUTSqol measures the influence of urinary incontinence problems on the quality of life, and changes in interpersonal relations in everyday life [57].

##### Secondary exploratory outcomes

- Fear of falling: Falls Efficacy Scale International (FES-I) [58, 59]. The FES-I is a patient-reported outcome that measures the level of concern about falling during daily activities inside and outside the home whether or not she actually does the activity [58, 59]. This 16-item questionnaire has a range of 16–64. Higher scores indicate lower fall efficacy. Its target population is older adults with or without a history of falling. It is responsive to change with good predictive validity for future falls, muscle weakness, and overall disability [60–63]. All items are highly relevant to the single underlying factor representing concern with falling [64]. It also has validated thresholds of clinical importance to older adults with a cut-point of ≥ 20 identified as at least a moderate level of concern.
- General satisfaction with performing one’s usual social roles and activities: PROMIS Satisfaction with Participation in Social Roles [23].
- Fall history: Incident falls and fall-related injuries over the 6 months prior to enrollment into the study [65].
- Fall incidence: Self-reported falls and fall-related injuries that occur during the 6-month study period.
- Lower body functioning: Short Physical Performance Battery (SPPB) [66, 67]. The SPPB includes three objective tests of lower body function: (1) a timed 12-foot walk; (2) 5 timed, repetitive chair stands; and (3) a hierarchical test of standing balance. A rank ordinal score is given for each test, then all 3 are summed. SPPB total scores range from 0 (worst performance) to 12 (best performance).
- Self-report physical functioning: PROMIS physical function 8b [68, 69]. Assesses self-reported capability rather than actual performance. This includes the functioning of upper and lower extremities, mobility, as well as instrumental activities of daily living, such as running errands [68, 69].
- Physical activity assessment: Physical activity monitoring via accelerometry using the Fitbit (Fitbit, San Francisco CA) will be used to assess sedentary and physical activity behaviors. Participants will be provided with a Fitbit consumer activity monitor to wear for the duration of the study. Participants will use the device as a simple step monitor and will be required to simply wear and charge the device periodically, and as such requires little technical skill. The Fitbit will be worn for 1 week immediately after randomization; for 1 week at the end of the 12-week intervention; and for 1 week at the 6-month follow-up. Participants will be provided with a postage-paid package so they can return the Fitbit to the research office at the end of each 1-week of activity monitoring. Participant data will be exported via the Fitbit website and retained for future exploratory analyses.

##### Sociodemographic and clinical variables

We will collect baseline sociodemographic and clinical data using standardized, validated instruments to be used for subgroup analyses. These data sources are well validated using the data collection approaches that the study will use. These include age; ethnicity; marital status; education level; incontinence type (urgency Ui, OAB, mixed UI) [70, 71]; comorbidities (Functional Comorbidity Index [72, 73]); medications [74] (name, dose, frequency of use of prescribed and over-the-counter medications, herbs, and supplements, total number of medications and medications known to affect bladder symptoms); past treatment experience; polypharmacy (≥ 5 routine medications (yes vs. no)) [75].

##### Structured qualitative interviews

Semi-structured interview information will be combined with quantitative trial results to explore participants’ lived experiences with these conditions; perceived benefits and harms of the interventions; and examine factors associated with treatment benefits and harms.

## Methods against bias

### Randomization

We will create an online password-protected randomization system that will be used to facilitate the random assignment of participants to study arms. A simple block randomization method will be used for this feasibility study to help ensure roughly balanced sample sizes across the three arms as the study progresses. This will allow us to make a more informed assessment of the overall feasibility of this design. The system will be created using the built-in randomization facility of the REDCap platform to be used for data entry. When the website is entered, the user will be asked to complete information that establishes subject eligibility. The group assignment will be revealed only if all eligibility criteria are satisfied.

### Blinding

It is not feasible to mask participants or clinicians due to the nature of the PFMT intervention. Our primary outcomes are obtained from validated self-report questionnaires reducing the possibility of observer bias. In addition, the outcome assessors will be blinded to ensure unbiased outcome findings.

### Sample size

The purpose of this project is to test the feasibility of our procedures, ability to recruit and enroll this patient population, test study the materials, and then to refine them [76, 77]. A total of *n* = 48 patients will be individually randomized (1:1:1) to PFMT, Mirabegron, and Trospium groups (16 in each arm). For the subsequent larger study to be feasible, > 80% of patients enrolled will need to be evaluable and > 70% assigned to each intervention must be adherent to the intervention. Forty-eight participants will allow us to decide whether the proportion of subjects being evaluable, *P*_1_, is ≤ 0.65 or ≥ 0.79. If the number being evaluable is ≥ 36, the hypothesis that *P*_1_ ≤ 0.65 is rejected with a target error rate of 0.1 and an actual error rate of 0.09. If the number of subjects being evaluable is ≤ 35, the hypothesis that *P*_1_ ≥ 0.79 is rejected with a target error rate of 0.20 and an actual error rate of 0.19. We would then consider evaluability successful if the number evaluable is ≥ 36 and we would consider it unsuccessful otherwise. Forty-eight will also allow us to decide whether the proportion who adhere to the protocol, *P*_2_, is ≤ 0.61 or ≥ 0.75. If the number who adhere ≥ 34, the hypothesis that *P*_2_ ≤ 0.61 is rejected with a target error rate of 0.1 and an actual error rate of 0.09. If the number is ≤ 33, the hypothesis that *P*_2_ ≥ 0.75 is rejected with a target error rate of 0.2 and an actual error rate of 0.19. We would then consider adherence successful if the number of subjects evaluated equals or exceeds 34 and we would consider it unsuccessful otherwise. Probability calculations were done using PASS 2021 software.

### Data management

REDCap will be used for all data entry. REDCap provides secure, web-based applications with an intuitive interface for users to enter data with real-time validation rules such as range checks for categorical and continuous variables, automatic branching logic, checks for internal consistency within a form, and calculation of derived variables.

### Data security and confidentiality

Important features of REDCap and standard procedures of the Division of Biostatistics at UTMB provide a high degree of certainty that data will never be lost and that subject confidentiality will be maintained. In accordance with the two key requirements of HIPAA, password protection will be required both for access to study computers and REDCap. Only authorized personnel will be given access to the data entry system and those personnel will only be able to access data from their own clinic site. All web-based information transmission from and to REDCap is encrypted. Standard security and confidentiality measures at UTMB include the requirement that employees sign confidentiality agreements, personal identifiers are included in electronic databases only under strong necessity, and encryption is used when names, addresses, and other primary identifiers are present in SAS datasets. Access to all computers and to REDCap is automatically logged. Should problems arise with the system, a full-time, highly qualified network engineer runs the system and is on call 24 h a day, 7 days a week.

### Data safety and monitoring plan

This study has a DSMP stand-alone document that is not included in the protocol document.

## Statistical analysis

### Analysis plan

#### Aim 1

Feasibility will be analyzed using descriptive statistics (frequencies, means, and standard deviations) and evaluated based on a successful evaluable and adherence rate. The study will be considered not feasible if ≤ 35/48 participants are evaluable or if ≤ 33/48 are adherent. Acceptability, fidelity, and safety will all be analyzed with descriptive statistics as well and these parameters will also inform the decision to move forward. We will perform an intention-to-treat analysis and include all participants who have been randomized to their intended treatment arm irrespective of adherence to the study protocol. As a secondary analysis, we will run one-sided exact tests to assess whether sample proportions of evaluability and adherence warrant dropping an arm in a future study.

#### Aim 2

This aim is exploratory and will provide estimates for UI and fall-related outcomes in each arm. Continuous outcomes will be compared between treatment arms using the Wilcoxon Rank Sum test. For categorical outcomes, a Fisher’s exact test will be used to compare rates between treatment arms. These comparisons are likely underpowered, and this Aim is not designed to make conclusions about comparative effectiveness but will provide preliminary estimates to inform the larger study.

#### Aim 3

The semi-structured interviews will be digitally recorded and professionally transcribed. We will use a 6-stage iterative, thematic analysis approach [78]. The thematic framework will be developed using NVivo version 12 Plus. A thematic analysis network will be constructed based on the recurring themes identified. Quantitative and qualitative findings will then be triangulated by identifying corroborated or divergent findings [79]. Integration of quantitative and qualitative data will allow us to identify treatment perceptions that go beyond a pre-determined point reduction on the outcome measures, as well as to identify additional facilitators and barriers to treatment response and better support insight into the implementation of findings into clinical practice.

## Discussion

This feasibility study is a randomized, multi-arm, mixed methods, clinical trial comparing three currently in use initial treatment options for OAB in older women who also screen positive for increased risk of falling: (1) a 12-week structured behaviorally based pelvic floor muscle training (PFMT) intervention administered by physical therapists in the clinic; (2) a 12-week course of the beta-3 agonist, Mirabegron; and (3) a 12-week course of the antimuscarinic, trospium chloride. Behavioral interventions and medications elicit their effects through very different mechanisms of action that may influence fall-related outcomes differently. There is also a critical lack of direct head-to-head trial evidence on non-pharmacologic and pharmacologic treatments in this patient population [5] — particularly in women with co-existing conditions, including fall risk [6]. Study feasibility will be determined through key milestones on evaluability, adherence to the interventions, attrition, adverse events, productive recruitment methods, and sample characteristics. We will also measure important indicators of symptom severity, quality of life, physical activity, falls, and fear of falling. The proposed study is the first to compare these common non-surgical treatments for UUI and OAB in a high fall risk patient population and will lay the groundwork for a program of research investigating the bidirectional relationships that exist across these two common geriatric syndromes both at the level of shared risk factors and response to treatment.

## Data Availability

This is a protocol paper, and no data are available yet. De-identified data, assessment and intervention materials, and analytic code from this study will be made available with subsequent papers.
